# Photoperiodic regime influences onset of lens opacities in a non-human primate

**DOI:** 10.7717/peerj.3258

**Published:** 2017-05-04

**Authors:** Marko Dubicanac, Julia Strueve, Nadine Mestre-Frances, Jean-Michel Verdier, Elke Zimmermann, Marine Joly

**Affiliations:** 1 Institute of Zoology, Tierärztliche Hochschule Hannover, Hanover, Lower Saxony, Germany; 2 Clinic for Small Animals, Tierärztliche Hochschule Hannover, Hanover, Lower Saxony, Germany; 3 Department of Molecular Mechanisms in Neurodegenerative Diseases Inserm U1198, Univ. Montpellier, Montpellier, France; 4 Centre for Comparative and Evolutionary Psychology, University of Portsmouth, Portsmouth, United Kingdom

**Keywords:** Nuclear sclerosis, Aging, Photoperiod, Mouse lemur, Cataract, Primate

## Abstract

**Background:**

Opacities of the lens are typical age-related phenomena which have a high influence on photoreception and consequently circadian rhythm. In mouse lemurs, a small bodied non-human primate, a high incidence (more than 50% when >seven years) of cataracts has been previously described during aging. Previous studies showed that photoperiodically induced accelerated annual rhythms alter some of mouse lemurs’ life history traits. Whether a modification of photoperiod also affects the onset of age dependent lens opacities has not been investigated so far. The aim of this study was therefore to characterise the type of opacity and the mouse lemurs’ age at its onset in two colonies with different photoperiodic regimen.

**Methods:**

Two of the largest mouse lemur colonies in Europe were investigated: Colony 1 having a natural annual photoperiodic regime and Colony 2 with an induced accelerated annual cycle. A slit-lamp was used to determine opacities in the lens. Furthermore, a subset of all animals which showed no opacities in the lens nucleus in the first examination but developed first changes in the following examination were further examined to estimate the age at onset of opacities. In total, 387 animals were examined and 57 represented the subset for age at onset estimation.

**Results:**

The first and most commonly observable opacity in the lens was nuclear sclerosis. Mouse lemurs from Colony 1 showed a delayed onset of nuclear sclerosis compared to mouse lemurs from Colony 2 (4.35 ± 1.50 years *vs.* 2.75 ± 0.99 years). For colony 1, the chronological age was equivalent to the number of seasonal cycles experienced by the mouse lemurs. For colony 2, in which seasonal cycles were accelerated by a factor of 1.5, mouse lemurs had experienced 4.13 ± 1.50 seasonal cycles in 2.75 ± 0.99 chronological years.

**Discussion:**

Our study showed clear differences in age at the onset of nuclear sclerosis formation between lemurs kept under different photoperiodic regimes. Instead of measuring the chronological age, the number of seasonal cycles (*N* = four) experienced by a mouse lemur can be used to estimate the risk of beginning nuclear sclerosis formation. Ophthalmological examinations should be taken into account when animals older than 5–6 seasonal cycles are used for experiments in which unrestricted visual ability has to be ensured. This study is the first to assess and demonstrate the influence of annual photoperiod regime on the incidence of lens opacities in a non-human primate.

## Introduction

Opacities of the lens are typical age-dependent pathologies having high impact on circadian rhythm in humans (e.g., Turner & Mainster, 2008; Turner, Someren & Mainster, 2010). Cataracts represent the most common cause for visual restriction in elderly humans (Pascolini & Mariotti, 2012). Cataract is an umbrella term that describes opacifications in the lens. The opacity can eventually occupy the whole lens and highly impacts eyesight which can result in complete blindness. Different forms of cataracts can be caused by a huge variety of reasons like age (Truscott, 2005; Vinson, 2006; Yoon, Kim & Shin, 2015; Žorić, Miric & Kisic, 2015), radiation (Cruickshanks, Klein & Klein, 1992; Delcourt et al., 2000; Tang et al., 2015), malnutrition (Heseker, 1995; Ohta et al., 1997; Meyer & Sekundo, 2005; Nourmohammadi et al., 2008; Yonova-Doing et al., 2016) or metabolic diseases (Miglior et al., 1994; Klein et al., 1995; McCarty et al., 1999; Gelatt, Gilger & Kern, 2012; Maggs, Miller & Ofri, 2012).

A typical manifestation in the lens is age-related nuclear cataract (ARN cataract) which starts forming in the centre until it finally involves all of the lens fibres. Oxidation seems to play a central role in the pathogenesis of this special form (Truscott, 2005; Vinson, 2006; Yoon, Kim & Shin, 2015; Žorić, Miric & Kisic, 2015). Antioxidants, especially reduced glutathione (GSH), are important defense-mechanisms which protect the lens proteins from oxidation. A lens nucleus that is affected by a cataract shows significantly lowered concentrations of GSH. This may be due to the aging lens which starts to form a barrier around the nucleus. This barrier is presumably caused by the uncoupling of gap junctions between mature fibre cells. Nevertheless, more studies are necessary to clarify this presumption, see Sweeney & Truscott (1998); Fan, Monnier & Whitson (2016). This barrier prevents GSH and other antioxidants from entering the core (Sweeney & Truscott, 1998; Moffat et al., 1999; Truscott, 2005). Since the concentration of GSH in the nucleus is lowered, more oxidized proteins (e.g., methionine residues become oxidised to methionine sulfoxide) in the lens core start to accumulate and form light scattering structures which prevent light from reaching the retina, finally occupying the whole lens and causing total blindness.

An important differential diagnosis to ARN cataract is nuclear sclerosis (NS). Nuclear sclerosis is caused by an increased density of lens fibers in the lens core and represents a physiological process of the aging lens (Gelatt, Gilger & Kern, 2012). Although NS is not known to cause blindness it may cause farsightedness and is playing a part in the formation of presbyopia (Strenk, Strenk & Koretz, 2005; Gelatt, Gilger & Kern, 2012). Since the lens capsule cannot expand, the lens fibers are compressed within the lens nucleus, this finally leading to visual opacities, loss of lens elasticity and presbyopia (Strenk, Strenk & Koretz, 2005; Gelatt, Gilger & Kern, 2012; Maggs, Miller & Ofri, 2012; Gelatt, 2013). The genetic background is still not clear. Nevertheless, studies suggest a polygenetic and environmental impact on familial aggregation instead of one major gene in humans (Klein et al., 2005). Nuclear sclerosis allows the examination of the retina when using indirect ophthalmoscopy while ARN cataract blocks the line of sight during indirect ophthalmoscopy and therefore, the two conditions can optically be differentiated from one another.

Cataracts may potentially occur in all species which accommodate using a lens and are not only frequently found in the human lens but are also commonly described in various animals like dogs (Gelatt, Gilger & Kern, 2012; Gelatt, 2013), horses (Matthews, 2000), non-human primates like macaques (Sasaki et al., 2011) and also frequently in the grey mouse lemur (Beltran et al., 2007). The grey mouse lemur belongs to the smallest primates worldwide (Mittermeier et al., 2010). Mouse lemurs are nocturnal and therefore have developed relatively large eye sizes (9.4 mm in diameter) compared to their skull (Kirk, 2004; Ross & Kirk, 2007). The combination of a relatively large eye size and higher life expectancy in captivity makes mouse lemurs highly prone to eye diseases. A whole variety of diseases have already been determined (Beltran et al., 2007), as for example corneal degeneration and dystrophy, pupil seclusion and most frequently cataracts. In animals older than seven years, cataracts were diagnosed in more than 50% of all investigated animals by Beltran et al. Although incipient anterior and posterior subcapsular cataracts were the most frequent findings, all stages of progression were observable (incipient, immature, mature and hypermature). The lack of adequate animal models in cataract research and the high incidence of cataracts in mouse lemurs make these animals a highly interesting model (Truscott, 2011).

Nowadays the grey mouse lemur (*Microcebus murinus*) is suggested to represent a promising non-human primate model in aging (Perret, 1997; Cayetanot et al., 2005; Gomez et al., 2012; Languille et al., 2012; Zimmermann & Radespiel, 2014; Zimmermann et al., 2016) and Alzheimer’s research (Austad & Fischer, 2011; Verdier et al., 2015). With a life expectancy of about eight years in the wild (Zimmermann et al., 2016) and up to 18.5 years (Weigl & Jones, 2005) in captivity, mouse lemurs live much shorter than other non-human primates. Most notable are deficiencies in behaviour and cognition (Nemoz-Bertholet & Aujard, 2003; Joly, Deputte & Verdier, 2006; Trouche et al., 2010; Joly et al., 2014), aggregation of abnormal phosphorylated tau protein (Bons et al., 1995) and ß-amyloid plaques (Mestre-Frances et al., 2000) as well as cerebral atrophy (Dhenain et al., 2000; Kraska et al., 2011). Mouse lemurs are also at the centre of interest for evolutionary research since they show highly flexible adaptations to their natural habitats and a high cryptic diversity between species (Zimmermann & Radespiel, 2014). The Broad Institute has also recently sequenced the genome of mouse lemurs (GenBank accession number ABDC00000000).

The photoperiod has a major impact on the annual rhythm of mouse lemurs regarding physiological constitutions like body weight, locomotion, lifespan and sexual function (Perret, 1997; Cayetanot et al., 2005) or life history patterns such as female body mass at first reproduction, female age at first reproduction as well as longevity (Zimmermann et al., 2016). As long-day breeders, mouse lemurs breed when day-length oversteps 12 h of sunlight (rainy season/summertime on Madagascar) which applies to six months out of 12 months per year under natural conditions (Perret, 1997). Under artificially accelerated light conditions, the non-breeding as well as the breeding season can be shortened to a total amount of eight months which equally accelerates the reproductive capability. This physiological characteristic revealed an interesting peculiarity in the aging mechanism in mouse lemurs. Animals held under accelerated photoperiodic conditions age faster and show typical age related symptoms earlier, e.g., grey fur around the eyes and flattening of the snout as well as age-dependent pathologies like cataracts (Perret, 1997; Dubicanac et al., 2014). Furthermore, they have lower body weight, show locomotion activity patterns resembling those of aged mouse lemurs, have a shortened lifespan equivalent to the shortened photoperiodic year and males show earlier sexual activity (Perret, 1997; Cayetanot et al., 2005). This dependency is due to the alternation of periods of dry and wet season which strictly dictates breeding seasons in these parts of Madagascar. The age of these animals therefore seems to be based on the numbers of seasonal cycles instead of chronological age (Perret, 1997).

The main aim of this study was to characterise the development of lens opacities and to compare the age at onset in two colonies with different photoperiodic regimes. Since it was described that mouse lemurs age faster when kept under accelerated photoperiodic cycles, we hypothesise that animals kept under accelerated photoperiodic cycles should develop age-related cataracts and/or nuclear sclerosis earlier than animals kept under a normal photoperiodic regime.

## Material & Methods

### Animals and maintenance

We examined mouse lemurs (*Microcebus murinus*) housed in two licensed breeding colonies kept under different photoperiodic regimes at the Institute of Zoology at the University of Veterinary Medicine Hannover, Germany (for details regarding housing conditions see (Wrogemann, Radespiel & Zimmermann, 2001); Hannover breeding license number 42500/1H) and at the University of Montpellier 2, France (Agreement No. ≠C-34-172-23). The animals were kept in cages with up to four individuals at constant temperature and humidity, had unrestricted access to water and received fresh food (mix of fruits, vegetables, nuts and insects) each day. In both facilities all animals were born in captivity and kept under artificial light conditions with a reversed light cycle.

The photoperiodic regime in Hannover (Colony 1) was based on annual photoperiodic cycles on Madagascar. The photoperiodic year lasts 12 months (eight months long-day period and four months short-day period). In Montpellier (Colony 2) the photoperiodic regime was accelerated. Therefore, the photoperiodically triggered reproductive “year” lasted eight months (five months long-day period and three months short-day period) instead of 12 months. Studies show that aging processes in gray mouse lemurs can be accelerated by the factor 1.5 when kept under these conditions (Perret, 1997; Languille et al., 2012; Dubicanac et al., 2014; Zimmermann et al., 2016).

Colony 1 was investigated three times, between March and April in 2012, 2013 and 2014. Colony 2 was investigated twice in May 2012 and one year later, in May 2013. In total 387 animals were investigated, 100 animals in colony 1 (49 males, 51 females) and 287 animals in colony 2 (130 males, 157 females) ranging from three months to 13.6 years. To determine potential eye diseases each animal underwent an ophthalmological investigation.

### Ophthalmological investigation

The examinations in this purely observational study were all licensed by the respective authorities (Hannover licence number, 33.9-42502-05-11A200, LAVES to Elke Zimmermann; Montpellier license number, B-34-8 to Nadine Mestre-Frances) and comply with animal care regulations, the applicable national law and the legal requirements of both countries.

All animals were habituated to weekly handling procedures for health checks, thus minimising stress during the ophthalmological examination. The sleeping-boxes (equipped with a lockable door) were used for transportation from the animal cage to the examination room. All mouse lemurs got a complete eye examination which was conducted at the end of the sleeping period/beginning of the activity period.

First menace and pupillary light reflexes were tested. The intraocular pressure was measured with the TonoVet^®^ (TonoVet^®^; ICare, Finland Oy). A slit-lamp (SL-14; Kowa, Eickemeyer, Germany) was used to examine the cornea, anterior eye chamber and the iris. After inducing mydriasis with tropicamid (Mydrum^®^, Chauvin ankerpharm GmbH, Berlin, Germany) the lens was also examined with the slit-lamp. Then an indirect ophthalmoscopy (Omega 100; Heine, Ettenheim, Germany) was made to examine the retina. All ocular findings were noted down on a self-prepared testing sheet similar to those used in clinical ophthalmological examinations.

Special attention within the ophthalmological examinations was given to the slit-lamp examination. Each eye was scanned carefully to determine any kind of opacity within the lens. While any kind of opacity was noted and estimated in size, special attention was given to opacities in the centre of the lens. Therefore, all animals showing no opacities during the first investigation were given more attention when being reinvestigated in the following year.

### UV-light

UV-light emission was measured using a UV-light meter (UV LIGHT METER, YK-35UV, Lutron Electronic Enterprise Co., LTD., Taiwan). The measurable wavelength ranged from 290-390 nm and therefore included the UV-A/-B range. The measurement was carried out when the white light was turned on “day-time” as well as when the white light was turned off and the red light was turned on “night-time”. In both facilities no UV-light could be detected. The measured value at “day-time” as well as at “night-time” was zero for all rooms (W/m^2^ = 0).

### Data analysis

**Figure 1:**
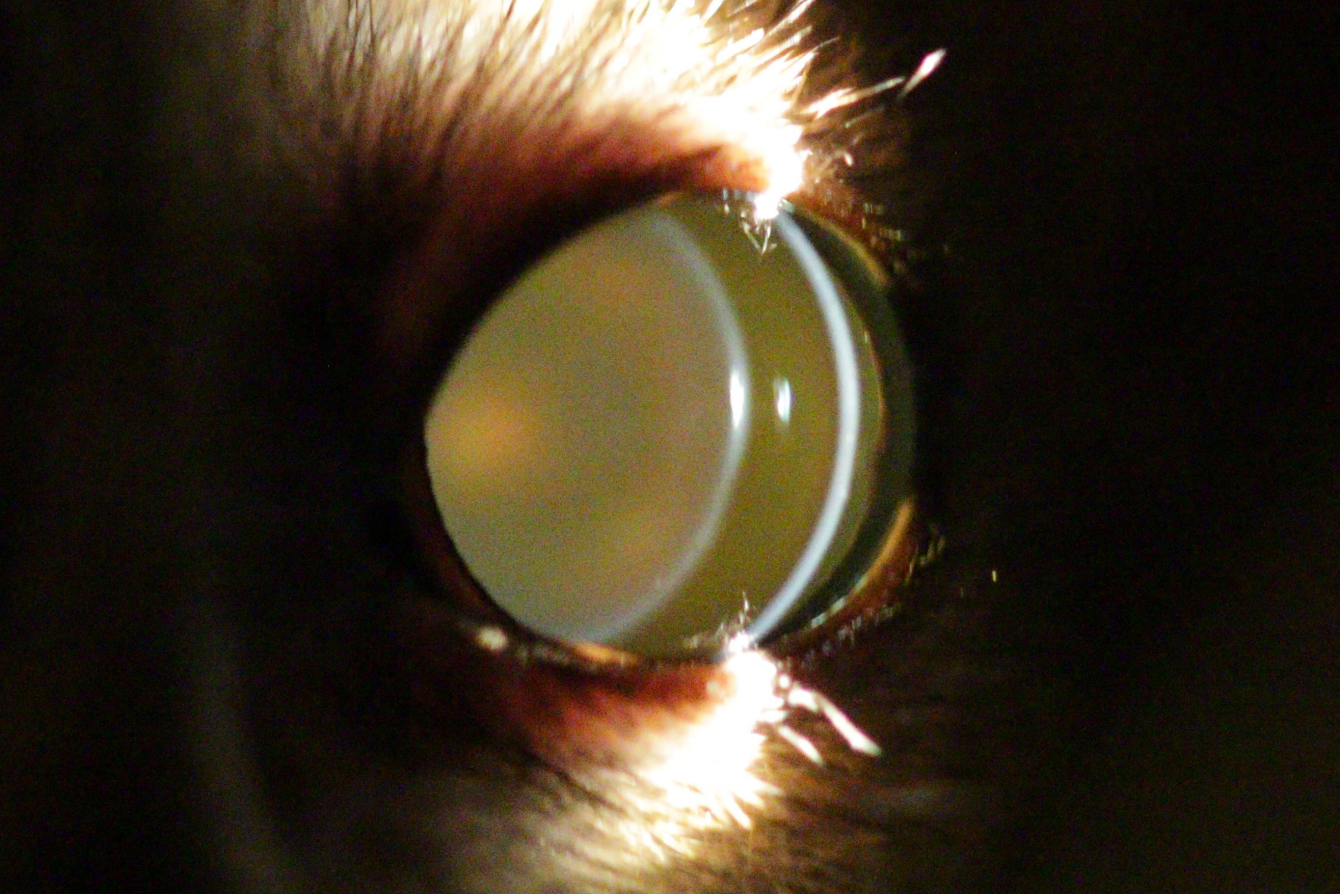
Eye of a two year old mouse lemur. This lens shows no opacities.

**Figure 2:**
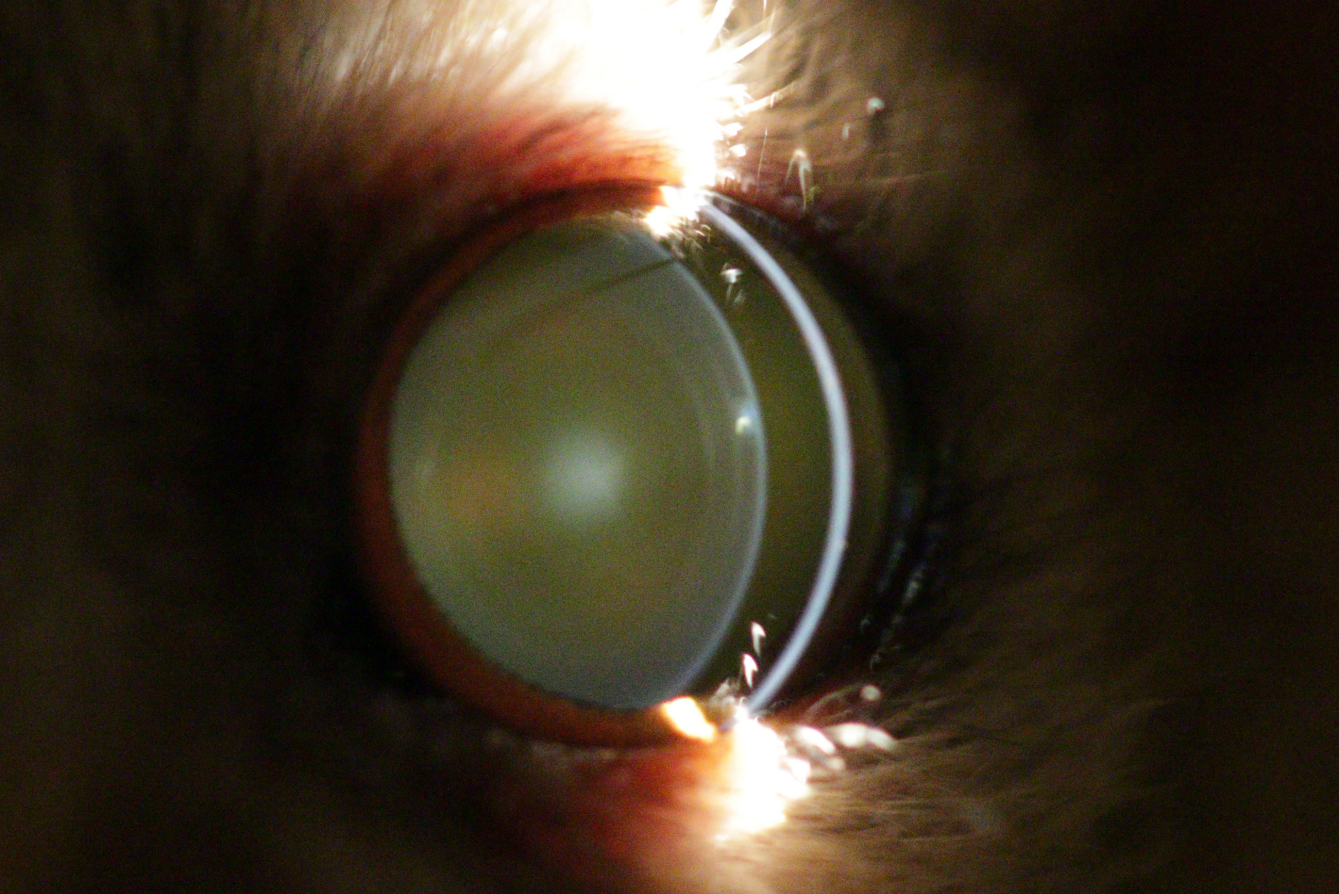
Eye of a four year old mouse lemur. The lens shows first signs of nuclear sclerosis in the center of the lens. Additionally incipient posterior cortical cataract is visible.

For each colony we determined the number of animals showing any kind of opacity and the age at its onset by selecting animals which showed no opacity in the first examination (see Fig. 1), but were positively tested in the second examination one year later (see Fig. 2). This subset consisted of 27 animals from colony 1 and 30 animals from colony 2 which all showed nuclear sclerosis as the first observable opacity. The age groups of both facilities were separately analysed for mean, range, standard deviation and median. Animals showing other pathologies than cataracts or nuclear sclerosis were excluded from the analysis. Findings for both colonies were compared using the Mann–Whitney-*U* test and either chronological age and or the number of seasonal cycles.

### Software for statistical analysis

All statistical analyses were performed using SPSS 23.0 for Windows. Significance level was set at *P* = 0.05.

## Results

### Overall diagnosed eye pathologies in both colonies

Fifty-one animals out of 100 (51%) in colony 1 and 192 animals out of 287 (66.9%) in colony 2 showed certain stages of cataracts and/or NS. (see Table 1) Out of these animals showing opacities, NS was the most frequent opacity with 45 out of 51 (88.2%) cases in colony 1 and 184 out of 192 (95.8%) cases in colony 2. It was followed by incipient anterior cortical cataracts with 28 out of 51 (54.9%) cases in colony 1 and 85 out of 192 (44.3%) cases in colony 2. Other less frequent findings were incipient posterior cortical cataracts (six cases in colony 1; 15 cases in colony 2), incipient anterior & posterior subcapsular cataracts (three cases in colony 1; six cases in colony 2), immature nuclear cataract (one case in colony 2) and mature cataracts (five cases in colony 1).

**Table 1:** Overview of cataract/NS incidences in both colonies. This table shows the total amount of animals positively tested for cataract and/or NS at any stage.

	Colony 1			Colony 2		
	Number of investigated animals	Number of animals with nuclear sclerosis	Number of animals with cataract	Number of investigated animals	Number of animals with nuclear sclerosis	Number of animals with cataract
Age (in years)	100	45	34	287	184	86
0–1	11	0	0	42	0	0
1–2	19	1	0	47	21	2
2–3	9	1	0	70	46	7
3–4	21	11	5	31	22	9
4–5	8	6	3	28	27	11
5–6	5	4	4	22	22	13
6–7	6	6	4	30	30	28
7–8	3	3	0	11	11	10
8–9	9	9	9	3	2	3
9–10	2	0	2	1	1	1
10–11	2	2	2	2	2	2
11–12	4	2	4	–		–
12–13	0	0	0	–		–
13–14	1	0	1	–		–

Other pathologies like ocular hypertension, synechia, corneal degeneration, hyphema, posterior lens luxation and phthisis bulbi were diagnosed sporadically in either one or both colonies.

### Cataracts and NS findings during aging

**Figure 3:**
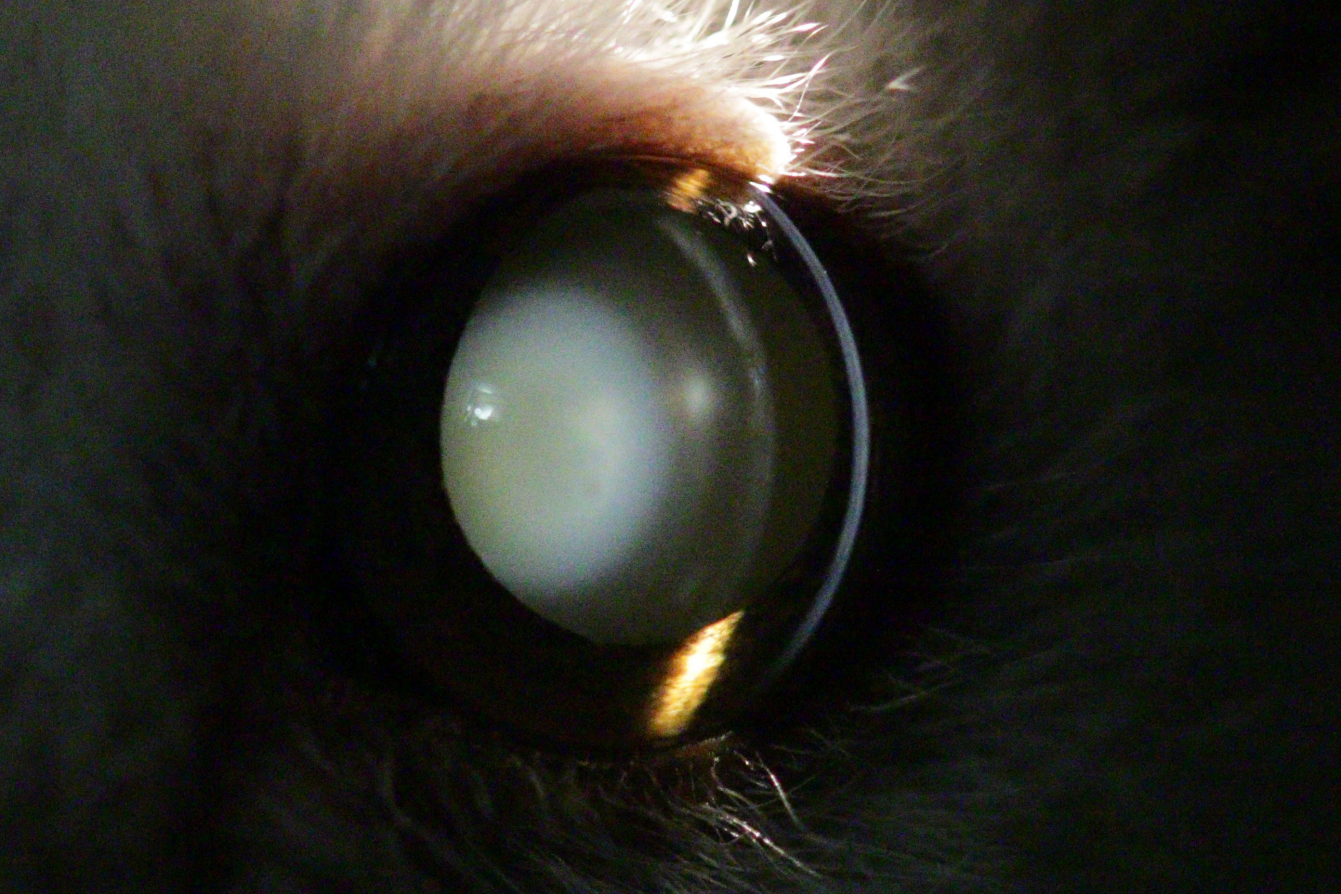
Eye of an eight year old mouse lemur. The lens shows immature, nuclear cataract with additional incipient anterior cortical cataract and anterior subcapsular cataract.

**Figure 4:**
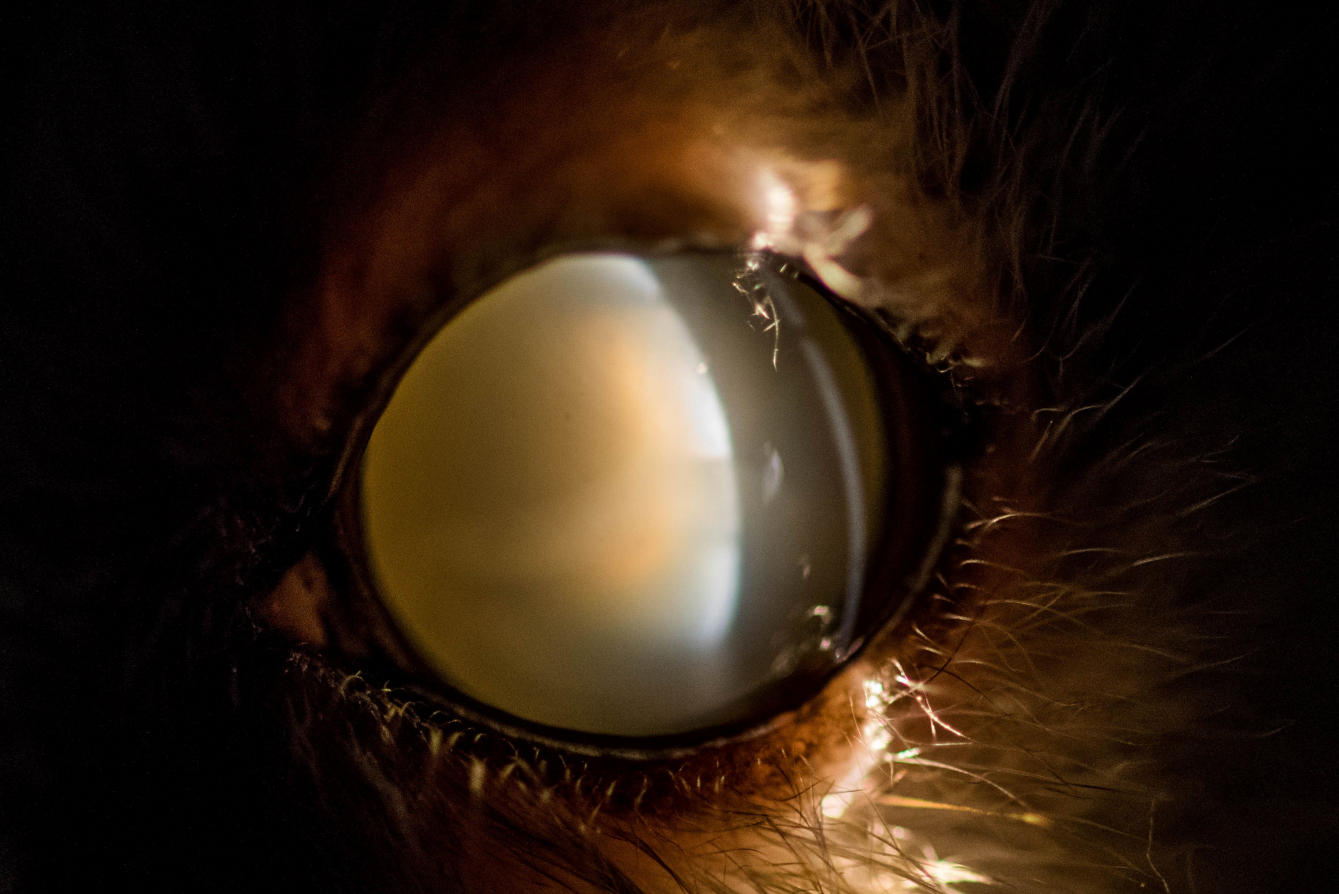
Eye of an 11 year old mouse lemur. The lens is affected by mature cataract.

The first visual opacity the examined mouse lemurs developed was nuclear sclerosis, which usually becomes denser with age. Indirect ophthalmic evaluation of the retina through this type of opacity is still possible (see Fig. 2). One animal (*n* = one; ≥eight years) was additionally affected by nuclear cataract and animals frequently showed cortical cataracts in addition to NS and/or nuclear cataracts (*n* = 107; ≥1.7 years; see Fig. 3). This type of opacity impedes complete fundic evaluation depending on the location and percent of lens involvement. Smaller to moderate sized lens opacities did not prevent a full fundic examination. In several animals mature cataracts could be observed (*n* = five; ≥10 years) (see Fig. 4).

### Onset of lens opacity in the colonies

In all animals nuclear sclerosis was the first observable opacity. In colony 1 the mean age of animals with first signs of nuclear sclerosis was 4.35 ± 1.50 years (the median was 3.9 years; the range was 1.8–7.9 years). In colony 2, the mean age was 2.75 ± 0.99 years (the median was 2.5 years; the range was 1.5–5.3 years). For colony 1, the chronological age was equivalent to the number of seasonal cycles experienced by the mouse lemurs. For colony 2, mouse lemurs had experienced 4.13 ± SD 1.50 cycles (median = 3.73 years; range = 2.2–8.0 years) before showing first signs of lens opacity.

**Figure 5:**
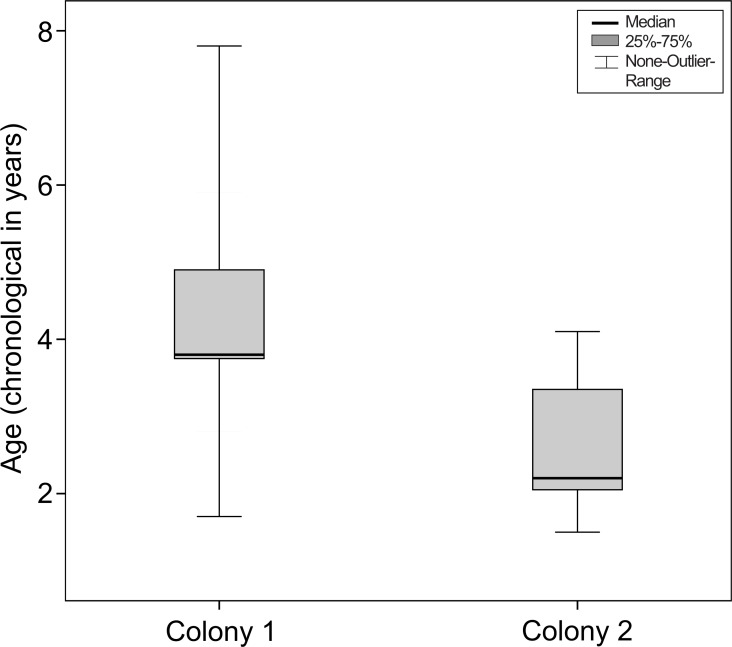
Age at the onset of nuclear sclerosis in mouse lemurs of two colonies with different photoperiodic cycles, measured in chronological years. Chronological age of mouse lemurs showing first signs of nuclear sclerosis in both investigated colonies. Nuclear sclerosis is present at a significant younger age in colony 2 than colony 1 (colony 1, *N* = 27 animals; colony 2, *N* = 30 animals; *P* < 0.001).

Therefore, when taking into account their chronological age, mouse lemurs showing nuclear sclerosis for the first time were older in colony 1 than in colony 2 (Mann–Whitney-test, *N*_total_ = 57, *N*_colony1_ = 27, *N*_colony2_ = 30, *U* =  − 4.030, *P* < 0.001, see Fig. 5).

**Figure 6:**
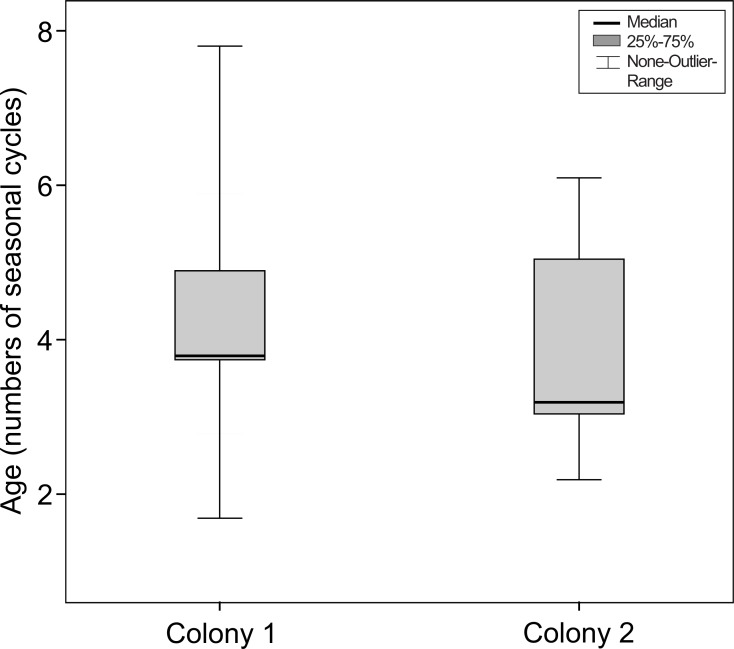
Age at the onset of nuclear sclerosis in mouse lemurs of two colonies with different photoperiodic cycles, measured in number of seasonal cycles. Number of seasonal cycles experienced by mouse lemurs showing first signs of nuclear sclerosis in both investigated colonies. The onset of NS does not significantly differ between both colonies (colony 1, *N* = 27 animals; colony 2, *N* = 30 animals; *P* = 0.671).

This difference between colonies did not remain if the number of seasonal cycles experienced by the mouse lemurs was considered (Mann–Whitney-test, *N*_total_ = 57, *N*_colony1_ = 27, *N*_colony2_ = 30, *U* =  − 0.424, *P* = 0.671, see Fig. 6).

## Discussion

### What was first, cataract or nuclear sclerosis?

In our present study we identified nuclear sclerosis as being the first age-dependent lens opacity. Indirect ophthalmological examinations of the retina through the opacity were possible in even more advanced stages of NS. The reason for complete blindness in old age on the other hand seems to be caused by forms of cataracts, which not allow an unrestricted view of the retina and have only been found in old animals.

Nuclear sclerosis starts to form in the nucleus of the lens which still allows an ophthalmologic examination of the retina. Nuclear sclerosis is caused by an increased density of lens fibers within the lens nucleus, this finally leading to visual opacities, loss of lens elasticity and presbyopia (Strenk, Strenk & Koretz, 2005; Gelatt, Gilger & Kern, 2012; Maggs, Miller & Ofri, 2012; Gelatt, 2013). Nuclear sclerosis in mouse lemurs seems to appear denser than expected when investigated by slit-lamp microscopy. Although it is quite easy to distinguish NS from a cataract by indirect ophthalmoscopy it may easily lead to misinterpretation of findings when using a slit-lamp alone. This presumption has to be evaluated by further histological examinations and represents the experience made by the investigator. However, mouse lemurs do show high incidence of progressive cataract formation in old age leading to complete blindness and are therefore unlikely to be affected by nuclear sclerosis alone. Nuclear sclerosis typically represents the first visible opacity in the lens of mouse lemurs, while middle-aged mouse lemurs may develop different types of cataracts (most frequently anterior subcortical cataracts) which regularly result in mature cataracts in old individuals (approx. ≥10 years).

Previous examinations in mouse lemurs performed by Beltran et al. (2007) in other colonies mention cataracts and not nuclear sclerosis as being the initial abnormality, which usually would be expected in aging eyes in other species (Beltran et al., 2007). We can partly confirm the high incidence of cataracts described in Beltran’s study. Nevertheless in our research NS was the most common and first visible opacity, while Beltran describes anterior and/or posterior subcapsular cataracts as being the most frequent and first visible opacity. It is unclear if Beltran did not diagnose NS at all or if diagnosed cases were categorised as physiological aging processes and consequently remain unmentioned in the list of ocular findings. It is worth stating that the overall incidence of cataracts in our investigated colonies seem to be higher (34% colony 1—median age: 3.9 years; 30% colony 2—median age: 2.8 years) than in colony 2 in Beltran’s study (21% colony 2—median age: four years) when the colonies are compared based on their chronological age. Both colonies in Beltran’s study were kept under similar light conditions as colony 1 in our own study (six months long-day and six months short-day). For example colony 1 in our study has a similar median age as colony 2 in Beltran’s work but an approximately 66% higher cataract incidence. The average age of colony 2 in our study is 1.2 years younger than the average age of colony 2 in Beltran’s study but has an approximately 43% higher cataract incidence. When the number of seasonal cycles is considered for colony 2 in our study (2.8 years × 1.5 = 4.2) instead of chronological age, the discrepancies to the compared colonies seem reduced. An explanation could be that the photoperiodic regime, similar to its influence on NS onset, may also influence the onset of cataracts. Nonetheless, further studies are required and a longitudinal study would be necessary to confirm this presumption. As the photoperiodic cycles are the same and UV light was ruled out in our study the discrepancies between colony 1 (34%—median age: 3.9 years) in our study and colony 2 (21%—median age: 4 years) in Beltran’s study could be based on differences in nutrition management and supply of antioxidants. Lastly, in the present study, due to time restrictions and animal availability, our examinations were conducted only annually. A more frequent interval of examinations would definitely help to determine with more accuracy the exact onset of cataracts or NS in mouse lemurs, which seems crucial for future studies.

### Nuclear sclerosis onset and its dependency on the photoperiod

In our study, mouse lemurs showing first signs of NS in colony 1 were significantly older than those from colony 2 (when chronological age is considered). The number of seasonal cycles experienced by mouse lemurs seems therefore to determine the onset of nuclear sclerosis. These results match well with our presumption that NS may be the cause of the initial opacity in the lens of mouse lemurs and that its onset depends more on photoperiodic cycles than on chronological age. As a physiological process NS onset is in line with other physiological aging effects, like grey fur around the eyes and flattening of the snout, which also show progression depending on photoperiodic cycles (Perret, 1997; Cayetanot et al., 2005; Languille et al., 2012). In humans, it is assumed that both environmental and polygenetic effects play a role in the etiology of NS (Klein et al., 2005). Our results in grey mouse lemurs point towards a photoperiod-dependent onset of NS. Like the acceleration of other physiological aging processes in the grey mouse lemur, the onset of NS is accelerated when photoperiodic cycles are shortened.

The effect of opacities in the lens on circadian photoreception and rhythm represents an important field in human research. In humans, crystalline lens opacities progressively increase with age causing a continual loss of circadian photoreception. Ten-year-old children have a circadian photoreception that is ten times higher than that of a 95-year-old human (Turner & Mainster, 2008). The loss of circadian photoreception highly affects the physiological and mental state and a diversity of cardiovascular, respiratory, endocrine, rheumatological and neurological diseases has been linked to variations in circadian rhythms (Klerman, 2005). A general loss of responsiveness to light has also been shown in aging mouse lemurs (Gomez et al., 2012). The relevant causes are still not clear but possible reasons could be similar to those in humans like the dysfunction of neural transmission or increased shortwave absorption of the lens (Jackson & Owsley, 2000; Revell & Skene, 2010). Although minor lens opacities were the most frequent finding in our study (like incipient cataract and beginning NS) and are not likely to have an effect on photoreception, it remains unclear which impact very dense opacities (mature cataract) have on the mouse lemurs’ photoreception. The aforementioned age-dependent loss of photoreception, the dense opacification of the lens in old individuals and the general dependency on photoperiodic cycles could influence one another, leading to severe pathophysiological changes similar to those in humans. Further investigations are necessary but could make this animal model interesting for a diversity of new medical research fields.

### Causes of cataracts which have to be considered and ruled out

Although nuclear sclerosis was the predominant opacity that we could observe, several forms of cataracts were found as well (incipient anterior cortical cataracts, incipient posterior cortical cataracts, incipient posterior/anterior subcapsular cataracts, immature nuclear cataract and mature cataracts). Usually the observed forms showed slow or no progression within the study period. Nevertheless, advanced stages of cataract occur in older individuals and have serious impact on the visual ability, which makes it necessary to rule out possible reasons.

UV-light seems to be predominantly associated with cortical cataract formation (Cruickshanks, Klein & Klein, 1992; Delcourt et al., 2000; Tang et al., 2015) but may potentially occur in any lens layer. Since no UV-light was detectable within the facilities where the lemurs were housed we can rule out this kind of radiation as an inducing factor.

Another important factor for cataract formation is diabetes mellitus (diabetes type 2). This kind of cataract usually shows fast progressive expansion within a few months (Basher & Roberts, 1995; Beam, Correa & Davidson, 1999; Li, Wan & Zhao, 2014) and is mainly associated with cortical cataracts (Miglior et al., 1994; Klein et al., 1995; McCarty et al., 1999; Li, Wan & Zhao, 2014). Although we could observe cortical cataracts frequently, slow or no progressive spreading was apparent in our study period. Therefore we conclude that the investigated lemurs were not affected by diabetic cataract forms.

It is unclear whether insufficient supply of antioxidative substances like vitamin E, C, B as well as essential amino acids such as tryptophan, phenylalanine, histidine and carotenoids may induce or accelerate cataract development (Heseker, 1995; Ohta et al., 1997; Meyer & Sekundo, 2005; Nourmohammadi et al., 2008). At least Vitamin C is described as being protective against both nuclear cataract formation and progression in humans (Yonova-Doing et al., 2016). In both facilities regular additions of vitamins and minerals were offered in mashed fruit mixtures to ensure sufficient supply. Nevertheless, the housing conditions of up to four animals in one cage do not ensure that each animal receives the same amount of food each day. Although cataracts were also frequently diagnosed in middle-aged animals with good body condition, age-related reduced absorption of nutrients (and consequently antioxidants) cannot be ruled out but seem unlikely. A possible explanation for these findings could be the fact that mouse lemurs go into torpor and reduce their metabolic rate during dry seasons (winter) on Madagascar. In captivity torpor is induced by the change of the photoperiodic regime from long-days to short-days. In case mouse lemurs suffer from malabsorption of antioxidative substances during this time period, oxidative processes in the lens may increase and promote the progression of cataracts. This topic has not been investigated so far and may be of interest for further studies.

## Conclusion

In our study, nuclear sclerosis represented the earliest stage and the most common opacity in the mouse lemurs’ lens. Here we showed clear differences in the onset of NS formation between two colonies kept under different photoperiodic regimes when measured in chronological age. The number of seasonal cycles experienced by the mouse lemurs was the main determinant for the onset of the lens opacities.

The number of seasonal cycles (*N* = four) experienced by a mouse lemur can be used to estimate the risk of beginning NS formation (approximately four years in colony 1 and approximately 3 years in colony 2) and for further studies which necessitate visual fitness in mouse lemurs. Ophthalmological examinations should be taken into account when animals older than 5–6 seasonal cycles are used for experiments in which unrestricted visual ability (e.g., unimpaired accommodation) has to be ensured. Due to the exceptionally high incidence of opacities in the lens of mouse lemurs, this is of utmost importance for further potential aging research studies.

##  Supplemental Information

10.7717/peerj.3258/supp-1 Supplemental Information 1 Animals with first signs of ocular opacities The tables show all animals from colony 1 and 2 which showed opacities in following examinations but had no visible opacities in the previous investigations. These are all animals which have been used for statistical analysis.

## List of abbreviations

ARN: cataracts: age-related nuclear cataracts
NS: nuclear sclerosis

## Additional Information and Declarations

### Data Availability

The following information was supplied regarding data availability: The raw data has been uploaded as a  Supplemental Information 1.
